# The impact of psychological capital on emergency and critical care nurses’ work engagement: the mediating effect of psychological resilience

**DOI:** 10.1590/1980-220X-REEUSP-2025-0290en

**Published:** 2026-04-03

**Authors:** Yuyan Wu, Kecai Xu, Peili Xu, Yefei Wang, Chaoliang Tang

**Affiliations:** 1 University of Science and Technology of China, Division of Life Sciences and Medicine, The First Affiliated Hospital of University of Science and Technology of China, Department of Anesthesiology, Hefei, Anhui Province, China.; 2 Anhui Provincial Cancer Hospital, Department of Anesthesiology, Hefei, Anhui Province, China.; 3 Anhui Medical University, The Third Affiliated Hospital of Anhui Medical University, Department of Nursing, Hefei, Anhui Province, China.; 4 AnHui Institute of Medicine, School of Nursing, Hefei, Anhui Province, China.

**Keywords:** Work Engagement, Resilience, Psychological, Mediation Analysis, Engajamento no Trabalho, Resiliência Psicológica, Análise de Mediação

## Abstract

**Objective:**

This study addresses this gap by examining how Psychological Capital directly impacts Work Engagement and how Psychological Resilience mediates this relationship among emergency and critical care nurses.

**Method:**

This study employed a cross-sectional survey design, utilizing random sampling to select 1,400 emergency and critical care nurses from 23 hospitals in China. Participants were asked to complete validated questionnaires measuring Psychological Capital, Psychological Resilience, and Work Engagement. Data were analyzed using Pearson’s correlation and structural equation modeling (SEM) to test the direct effects of Psychological Resilience and its mediating effects in the relationship between Psychological Capital and Work Engagement.

**Results:**

The results showed that Psychological Capital positively influences Work Engagement among emergency and critical care nurses. Additionally, Psychological Resilience plays a significant mediating role in this relationship, suggesting that nurses with higher Psychological Capital are more engaged in their work, partly due to their enhanced resilience.

**Conclusion:**

This study provides valuable insights for improving emergency and critical care nurses’ mental health and Work Engagement.

## RESUMO

**Objetivo:**

Este estudo aborda essa lacuna examinando como o Capital Psicológico impacta diretamente o Engajamento no Trabalho e como a Resiliência Psicológica medeia essa relação entre enfermeiros de emergência e terapia intensiva.

**Método:**

Este estudo empregou um delineamento transversal, utilizando amostragem aleatória para selecionar 1.400 enfermeiros de emergência e terapia intensiva de 23 hospitais na China. Os participantes foram solicitados a preencher questionários validados que mediam Capital Psicológico, Resiliência Psicológica e Engajamento no Trabalho. Os dados foram analisados utilizando a correlação de Pearson e modelagem de equações estruturais (MEE) para testar os efeitos diretos da Resiliência Psicológica e seus efeitos mediadores na relação entre Capital Psicológico e Engajamento no Trabalho.

**Resultados:**

Os resultados mostraram que o Capital Psicológico influencia positivamente o Engajamento no Trabalho entre enfermeiros de emergência e terapia intensiva. Além disso, a Resiliência Psicológica desempenha um papel mediador significativo nessa relação, sugerindo que enfermeiros com maior Capital Psicológico são mais engajados em seu trabalho, em parte devido à sua maior resiliência.

**Conclusão:**

Este estudo fornece informações valiosas para melhorar a saúde mental e o engajamento no trabalho de enfermeiros de emergência e de terapia intensiva.

## INTRODUCTION

Given the demanding nature of their work environment, nurses in emergency and intensive care units (ICUs) are constantly on the front lines of medical practice. Compared to their counterparts in other departments, they endure substantially higher levels of work-related stress, often leading to burnout and elevated turnover rates^(1)^. Psychological Capital comprises positive psychological traits such as self-efficacy, hope, and optimism, significantly impacting individuals’ behavior and performance^(2)^. Psychological Capital is considered an advanced psychological quality as a psychological resource and a positive psychological state that promotes personal development and performance improvement. Individuals with high Psychological Capital can withstand challenges, innovate, and transform adversity into opportunity, gradually achieving their ideals^(3)^. Studies have shown that Psychological Capital influences employees’ behavior and attitudes, determining their emotional intelligence and motivating them despite negative influences^(4)^.

Work Engagement is influenced by various factors, among which Psychological Capital, an emerging psychological concept, has received widespread attention in recent years^(5)^. Work Engagement is a positive, fulfilling emotional and cognitive state of mind characterized by vigor, dedication, and absorption^(6)^. It is a key predictor of nurses’ attitudes and behaviors^(7)^. Research^(8)^ indicates that critical care nurses in China exhibit moderate to low levels of Work Engagement. This insufficient engagement compromises the quality of nursing care and increases turnover intentions, posing a significant risk to the retention of skilled nursing professionals^(9)^.

Psychological Resilience refers to maintaining stable emotions and behaviors in the face of stress, challenges, and adversity, gradually adapting, coping, recovering, and even growing from these experiences. It includes trait-oriented, outcome-oriented, and process-oriented perspectives. Trait-oriented Resilience is an individual’s capacity to handle stress, setbacks, and adverse life events^(10)^. Outcome-oriented Resilience considers individuals who adapt well after high-risk experiences to have good Psychological Resilience^(11)^. Process-oriented Resilience emphasizes it as a dynamic process involving protective factors and individual differences when facing stress and setbacks^(12)^. Therefore, Psychological Resilience can help nurses maintain a positive mindset and high levels of Work Engagement when facing work challenges and stress^(13)^. Research suggests that nurses with strong Psychological Resilience are better equipped to handle adversity and trauma during public health emergencies, thanks to their ability to self-motivate^(14)^.

The study revealed that nurses’ job satisfaction, Psychological Capital, and Work Engagement were significantly interrelated, with Psychological Capital showing a strong and direct positive correlation with Work Engagement^(15)^.Extensive research has demonstrated a strong link between Psychological Capital and Psychological Resilience. Among newly recruited nurses, those with lower Psychological Capital but higher Psychological Resilience tend to exhibit greater adaptability in handling emergencies^(16)^. Moreover, interventions targeting Psychological Capital have been shown to significantly enhance the Psychological Resilience of ICU nurses, improve their emotional well-being, and alleviate burnout^(17)^.

Internationally, similar challenges have been reported. Emergency nurses in the United States frequently experience compassion fatigue and burnout due to high workloads and emotional demands^(18)^. In South Korea, depressive symptoms and turnover intention among nurses are closely associated with inadequate resilience and professional quality of life^(19)^. In European nursing research, higher Psychological Capital has been positively associated with greater Work Engagement and lower burnout, suggesting its protective role in nurses’ mental health and performance^(20)^. A systematic review further confirmed that resilience is a vital psychological resource that enables nurses to cope with stress and sustain engagement across different health care systems^(21)^. These findings highlight the global relevance of Psychological Capital and Psychological Resilience in shaping Work Engagement and reducing burnout among nurses.

The study examined the interconnections among nurses’ Work Engagement, Psychological Resilience, emotional labor, and burnout.The findings revealed a strong positive association between Work Engagement and Psychological Resilience. Based on these insights, increased Psychological Resilience training was recommended to foster higher Work Engagement and reduce burnout among nurses^(22)^.

**Figure 1:**
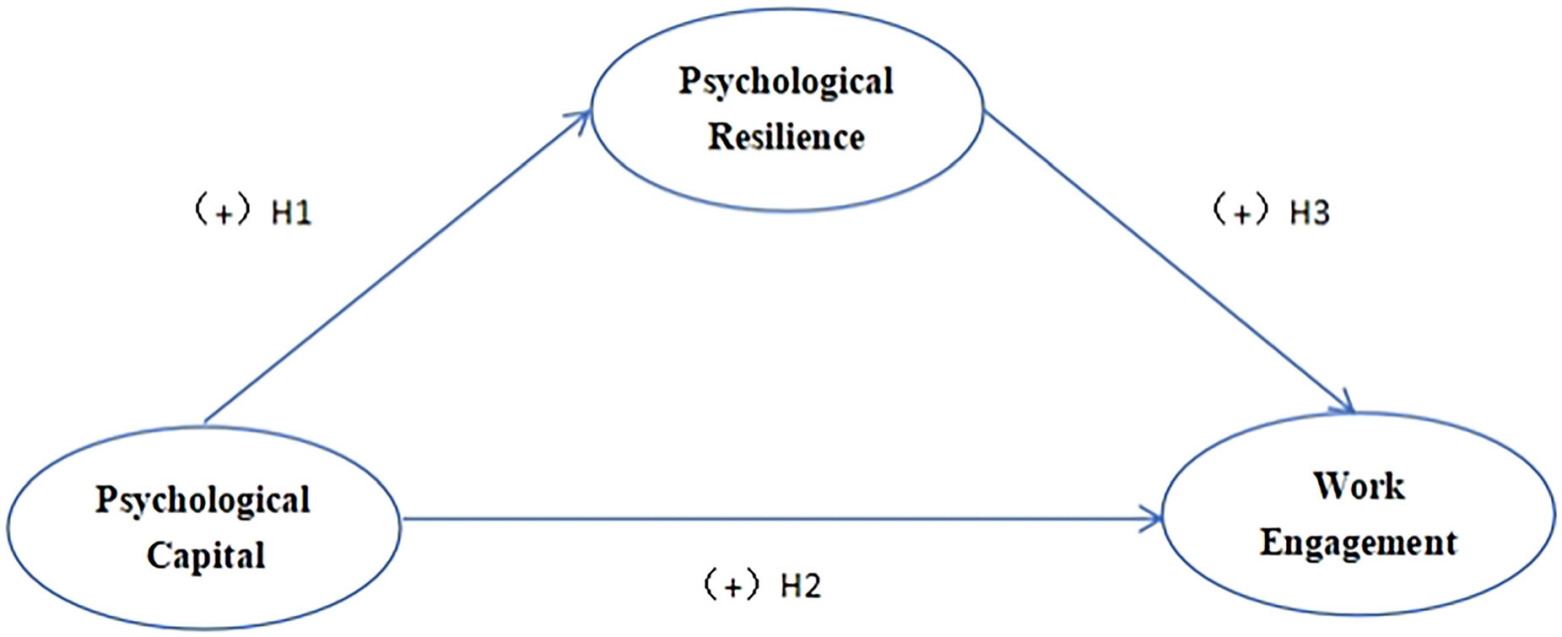
Hypotheses research model.

This study aims to answer the following research hypotheses: (1) The Psychological Capital influences Work Engagement among emergency and critical care nurses. (2) The Psychological Capital influences Psychological Resilience among emergency and critical care nurses. (3) Psychological Resilience influences Work Engagement among emergency and critical care nurses. (4) Psychological Resilience mediates the relationship between Psychological Capital and Work Engagement (Figure 1).

If these questions are answered, the findings may provide theoretical evidence for enhancing work engagement among emergency and critical care nurses and practical guidance for hospital administrators to develop appropriate interventions.

## METHODS

### Study Design

This study adopted a cross-sectional survey design, guided by the Strengthening the Reporting of Observational Studies in Epidemiology (STROBE) checklist, which is appropriate for observational cross-sectional studies.

### Study Setting

The study was conducted among emergency and critical care nurses employed in 23 hospitals across China, including both public and private institutions

### Participants and Sampling

The minimum required sample size was calculated using Slovin’s formula, $n=\frac{\text{N}}{{\text{1+Ne}}^{\text{2}}}$ at a 95% confidence level and a 5% margin of error^(23)^. With a finite population of *N* = 1,406 emergency and critical care nurses across 23 hospitals, the required minimum sample size was $n=\frac{\text{1406}}{\text{1+1406}\times \text{0}{\text{.05}}^{\text{2}}}\approx 311$ . To enhance representativeness, allow subgroup analyses by region and hospital type, and minimize non-response bias, a near-census strategy was adopted. Consequently, all eligible nurses across the 23 hospitals were invited to participate, and 1,400 valid responses were obtained (99.6% of the target population). Stratified random sampling ensured proportional representation by region and hospital type.

Inclusion criteria were: (1) age 18 years and above, (2) having at least one year of experience as an emergency and critical care nurse, and (3) informed consent and voluntary participation in the survey.

Exclusion criteria were: (1) inability to understand the questionnaire content correctly and (2) unwillingness to participate actively.

This study passed the ETHICS COMMITTEE OF HEFEI FIRST PEOPLE’s HOSPITAL, ERC code 2024-260-05.

### Procedures

In-service emergency and critical care nurses were selected as the survey subjects. The survey was conducted using the Questionnaire Star platform^(24)^. Before survey implementation, investigators received standardized training to ensure correct execution of the survey procedures and to address potential issues, thereby ensuring consistency in questionnaire administration. Prior to distributing the survey, the purpose of the study was clearly explained to the nurses, and informed consent was obtained, ensuring that participants understood the study and voluntarily participated. The survey was conducted at times that did not coincide with nurses’ busy periods, allowing sufficient time and energy for questionnaire completion. Patience and professionalism were maintained throughout the survey process, and any questions raised by nurses were addressed. The survey was designed to be completed within 20 minutes.

### Measures

#### Four Instruments Were Used

Four instruments were used in this study: a general information questionnaire, the Psychological Capital Scale, the Psychological Resilience Scale, and the Work Engagement Scale.

### General Information

The general information questionnaire was designed by the researchers and collected data on nurses’ gender, age, marital status, educational background, professional title, employing institution, and administrative positions.

### Psychological Capital

Psychological Capital was assessed using the Psychological Capital Scale, which includes four dimensions—optimism, resilience, hope, and self-efficacy—and comprises 20 items. The scale uses a six-point Likert format, with higher scores indicating higher levels of Psychological Capital. Each item is scored from 0 to 5, corresponding to “Very Disagree,” “Disagree,” “Slightly Disagree,” “Slightly Agree,” “Agree,” and “Very Much Agree.” The Cronbach’s alpha coefficients for the total scale and the four dimensions range from 0.718 to 0.923, and the test–retest reliability for the total scale is 0.819. The Chinese version of the scale has a Cronbach’s alpha coefficient of 0.898^(25)^. The scale demonstrated good construct validity (CMIN/DF = 13.86, CFI = 0.95, TLI = 0.94, RMSEA = 0.09, RMR = 0.05), with factor loadings for individual items exceeding 0.76. The average variance extracted (AVE) values for the four dimensions were 0.70, 0.85, 0.96, and 0.89.

### Psychological Resilience

Psychological Resilience was assessed using the Chinese version of the Connor–Davidson Resilience Scale (CD-RISC), which was translated and revised by Yu and Zhang^(26)^. The Chinese version of the CD-RISC consists of three dimensions—tenacity, strength, and optimism—and includes 25 items. It uses a five-point scoring system: 1 = “Never,” 2 = “Rarely,” 3 = “Sometimes,” 4 = “Often,” and 5 = “Always,” with a total score range of 25 to 125. Higher scores indicate better Psychological Resilience and greater stress resistance. The Cronbach’s alpha coefficient for the total scale is 0.91, indicating good reliability and validity and supporting its suitability for assessing the Psychological Resilience of nurse.

### Work Engagement

Work Engagement was assessed using the Chinese version of the Utrecht Work Engagement Scale (UWES-9)^(27)^. This scale is divided into three dimensions: vigor, dedication, and absorption, comprising nine items. Each item is scored from 0 to 6 (0 = never to 6 = always), with higher scores indicating higher engagement levels. The scale’s construct validity was assessed using exploratory factor analysis and confirmatory factor analysis. The UWES-9 demonstrates acceptable construct validity, with the three-factor model accounting for 57.76% of the variance and satisfactory fit statistics. The Cronbach’s alpha for the total scale is 0.93, with alpha values of 0.78, 0.80, and 0.81 for vigor, dedication, and absorption, respectively in Chinese populations.

### Quality Control

Strict quality control measures were implemented throughout the research process to ensure the scientific rigor and accuracy of the study results. During the research design phase, the research team conducted a literature review and consulted experts to gain an initial understanding of the Work Engagement of emergency and critical care nurses and its related influencing factors, thereby finalizing the study design. Appropriate research instruments were selected based on the study objectives, and a scientifically standardized survey plan was developed through multiple discussions.

During the formal survey phase, questionnaires were collected, checked, and organized immediately after the participants completed them. In the data organization phase, two individuals independently coded the valid questionnaires and double-checked the data to ensure accuracy. Following verification, the data were entered into Epidata 3.1 software^(28)^. After data entry, team members conducted logical checks on the values of each dimension of the evaluation indicators to ensure data accuracy and the reliability of the study results.

### Data Analysis

Descriptive statistical analyses of the study variables were performed using SPSS 24.0. Categorical variables were expressed as frequencies and percentages (*n*, %), while continuous variables were expressed as means ± standard deviations. Comparisons between two groups were conducted using independent samples *t* tests, and comparisons among multiple groups were conducted using analysis of variance (ANOVA). Pearson’s product-moment correlation coefficient was used to examine relationships among variables. Before conducting structural equation modeling (SEM), the Kaiser–Meyer–Olkin (KMO) measure and Bartlett’s test of sphericity were performed to assess the suitability of the data for factor analysis. SEM was used to examine the relationships among Psychological Capital, Work Engagement, and Psychological Resilience. SEM analyses were conducted using AMOS 22.0, allowing for the testing of both measurement and structural models. To ensure the robustness of the findings, bootstrapping with 5,000 resamples was employed to test the significance of indirect effects and mediation pathways, thereby confirming the reliability and validity of the observed mediation effects.

### Ethical Considerations

This study was approved by the Ethics Committee of Hefei First People’s Hospital (ERC code 2024-260-05). Participation was voluntary, informed consent was obtained, and confidentiality and anonymity were strictly maintained throughout the study.

## RESULTS

### Sample Characteristics

A total of 1400 valid questionnaires were obtained (effective response rate of 100%), with 707 female respondents (50.50%) and 693 male respondents (49.50%). Regarding the type of institution, 486 respondents (34.71%) were from private hospitals, and 914 respondents (65.29%) were from public hospitals. The professional titles of the respondents were as follows: 399 Nurses (28.50%), 442 Senior Nurses (31.57%), 543 Nurse Supervisors (38.79%), and 16 Associate Chief Nurses and above (1.14%). In the Chinese nursing system, nurses are primarily responsible for direct patient care and routine clinical tasks; senior nurses undertake more advanced clinical responsibilities and provide guidance to junior staff; nurse supervisors are responsible for managing wards or units, coordinating nursing teams, and ensuring quality of care; while associate chief nurses and above hold senior management roles, oversee nursing administration, education, and research, and contribute to policymaking. The age distribution was: 20−30 years old (595 respondents, 42.50%), 31−40 years old (443 respondents, 31.64%), 41−50 years old (329 respondents, 23.50%), and over 50 years old (33 respondents, 2.36%).

Regarding marital status, 1176 respondents (84.00%) were married, 189 respondents (13.50%) were unmarried, and 35 respondents (2.50%) were divorced. Also, 450 respondents (32.14%) did not hold administrative positions, while 950 respondents (67.86%) held administrative roles. As for the highest level of education, 592 respondents (42.29%) had a bachelor’s degree, 708 respondents (50.57%) had a master’s degree, and 100 respondents (7.14%) had a doctoral degree. The years of work experience were distributed as follows: 1−10 years (595 respondents, 42.50%), 11−20 years (443 respondents, 31.64%), and over 20 years (362 respondents, 25.86%).

### Reliability and Validity

Psychological Capital. The scale demonstrated good construct validity (CMIN/DF = 13.86, CFI = 0.95, TLI = 0.94, RMSEA = 0.09, RMR = 0.05), with factor loading of individual items above 0.76. The AVE values for the four dimensions were 0.70, 0.85, 0.96, and 0.89.

Psychological Resilience. The scale demonstrated good construct validity (CMIN/DF = 8.34, CFI = 0.95, TLI = 0.95, RMSEA = 0.07, RMR = 0.02), with factor loading of individual items above 0.69. The AVE values for the four dimensions were 0.74, 0.73, and 0.67.

Work Engagement. The scale demonstrated acceptable construct validity (CMIN/DF = 47.26, CFI = 0.98, TLI = 0.94, RMSEA = 0.18, RMR = 0.01), with factor loading of individual items above 0.90. The AVE values for the four dimensions were 0.76, 0.94, and 0.88.

### Harman’s Single-Factor Test Results

The questionnaire survey was conducted anonymously, including reverse-scored items to control for common method bias. Harman’s single-factor test examined the data for common method bias. The analysis included the primary variables of Psychological Capital, Work Engagement, and Psychological Resilience. The results indicated χ2/df = 13.24, CFI = 0.26, TLI = 0.34, and RMSEA = 0.27, suggesting no severe standard method bias in the study data,reinforcing the credibility of the study’s findings. The test revealed that no single factor emerged as dominant, suggesting that common method bias was not a major concern in this study.

### Current Status of Emergency and Critical Care Nurses’ Psychological Capital, Work Engagement, and Psychological Resilience

Descriptive statistical analyses of the study variables were performed using SPSS 24.0. Categorical data were expressed as frequency and percentage (*n*, %), while continuous data were expressed as mean ± standard deviation. Comparisons between two groups were conducted using the independent samples t-test, while comparisons among multiple groups were conducted using ANOVA. Pearson’s product-moment correlation coefficient was used to examine the relationships between variables. Before performing SEM, the Kaiser–Meyer–Olkin (KMO) measure and Bartlett’s test of sphericity were conducted to assess the adequacy of the data for factor analysis. The data were analyzed using SEM to examine the relationships among Psychological Capital, Work Engagement and Psychological Resilience. SEM was conducted using AMOS 22.0, which allowed for testing the measurement and structural models.

### Correlation Analysis

As shown in Table 1, the correlation analysis was conducted to examine the relationships between Psychological Capital, Work Engagement, and Psychological Resilience, using Pearson’s correlation coefficient to represent the strength of these relationships. The correlation coefficient between Psychological Capital and Work Engagement showed significance (*r* = 0.403, *p* < 0.01). The correlation coefficient between Psychological Capital and Psychological Resilience also showed significance (*r* = 0.540, *p* < 0.01). Similarly, the correlation coefficient between Work Engagement and Psychological Resilience showed significance (*r* = 0.415, *p* < 0.01), all indicating positive correlations.

**Table 1:** Correlation Analysis of Psychological Capital, Work Engagement, and Psychological Resilience Among emergency and critical care nurses — Hefei, Anhui, China, 2024.

	Psychological capital	Work engagement	Psychological resilience
Psychological Capital	1		
Work Engagement	0.403**	1	
Psychological Resilience	0.540**	0.415**	1

Note: *p < 0.05

**p < 0.01.

### Mediation Effect Test

This study employed SEM to test the mediating effect of Psychological Resilience in the relationship between Psychological Capital and Work Engagement among emergency and critical care nurses. The analysis was conducted using AMOS 22.0 software, applying the bias-corrected percentile Bootstrap method with 5,000 resamples to calculate the 95% confidence interval. The SEM analysis controlled for general information such as gender, age, and job title.

**Figure 2:**
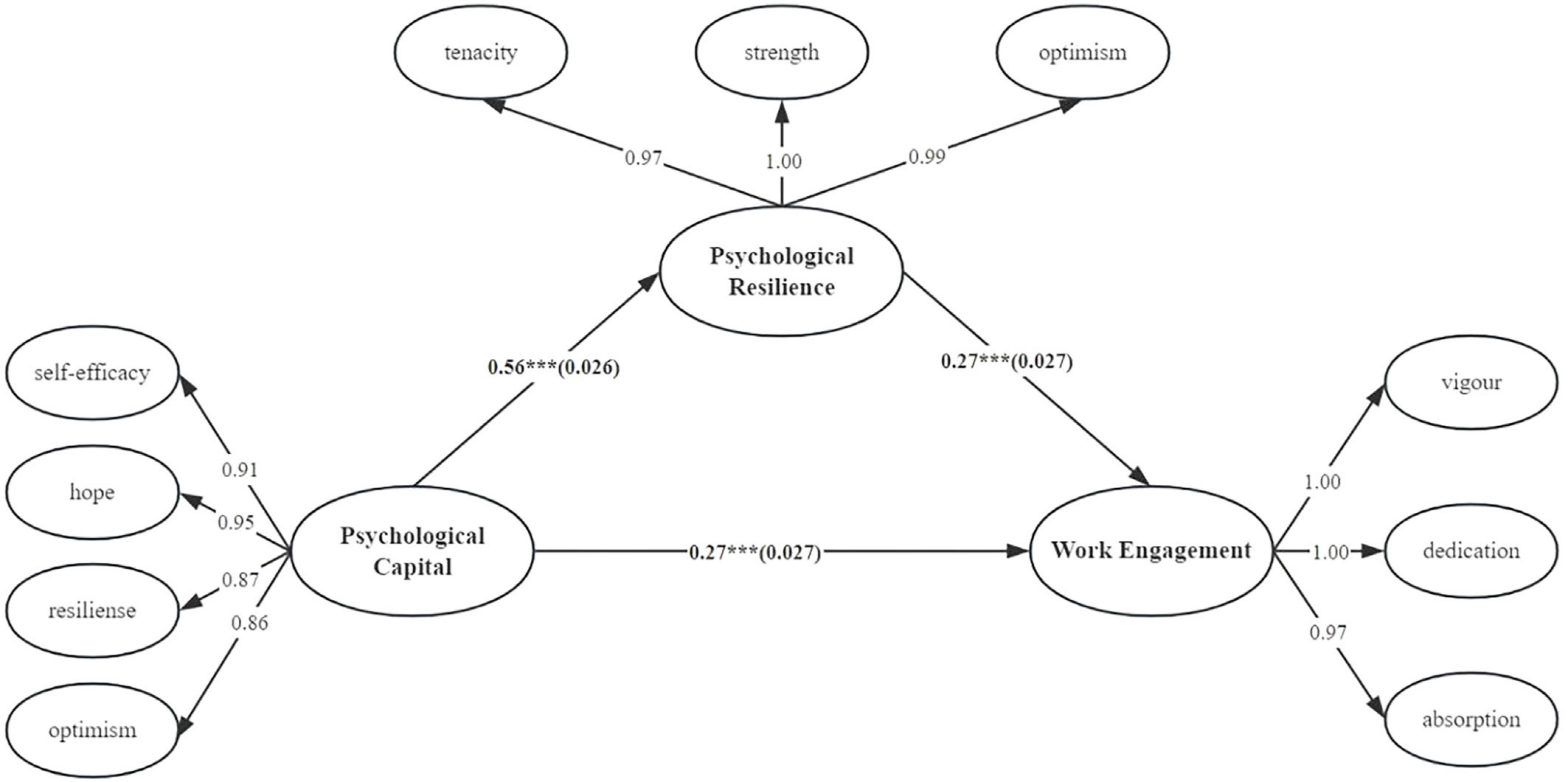
Results of mediation analysis (*n* = 1400).

Work Engagement was taken as the dependent variable, Psychological Capital as the independent variable, and Psychological Resilience as the mediating variable. Both direct and indirect effects were examined. The mediation model showed good fitness: CMIN/DF = 9.47, CFI = 0.90, TLI = 0.90, RMSEA = 0.08, RMR = 0.04. The standardized results of each regression path were presented in Figure 2. Psychological Capital positively predicted Psychological Resilience (β = 0.56, SE = 0.026, *p* < 0.001). Psychological Resilience positively predicted Work Engagement (β = 0.27, SE = 0.027, *p* < 0.001). Psychological Capital positively predicted Psychological Resilience (β = 0.27, SE = 0.027, *p* < 0.001). The results indicate that Psychological Resilience plays a significant partial mediating role in the relationship between Psychological Capital and Work Engagement.

The mediation effect analysis results (unstandardized) are shown in Table 2. The direct effect of Psychological Capital on Work Engagement is estimated at 0.233, with a 95% Bootstrap Confidence Interval ranging from 0.180 to 0.286. The indirect effect through mediator Psychological Resilience is 0.132, with the interval ranging from 0.091 to 0.173. The total impact combining direct and indirect paths is 0.365, with a confidence interval from 0.272 to 0.459. Notably, the mediation effect represents approximately 36.23% of the total impact, highlighting the significant role of Psychological Resilience in mediating the influence of Psychological Capital on Work Engagement.

**Table 2:** Summary of mediation effect test results — Hefei, Anhui, China, 2024.

	Value	95% LLCI	95% ULCI	Proportion
Direct effect	0.233	0.180	0.286	63.8%
Indirect effect	0.132	0.091	0.173	36.2%
Total effect	0.365	0.272	0.459	

Note: LLCI = lower level of confidence interval; ULCI = upper level of confidence interval.

## DISCUSSION

### Current Status and Relationships of Psychological Capital, Work Engagement, and Psychological Resilience of Emergency and Critical Care Nurses

This study explores the deeper relationship between Psychological Capital and Work Engagement among emergency and critical care nurses by considering Psychological Resilience as a mediating variable. The findings show a positive correlation between Psychological Capital, Work Engagement, and Psychological Resilience, suggesting that emergency and critical care nurses with higher Psychological Capital may also have higher Psychological Resilience and better Work Engagement. Psychological Capital is a psychological resource and positive psychological state that promotes personal development and performance enhancement. Psychological Capital belongs to advanced psychological qualities, including emotional and Psychological Resilience. Those with vital Psychological Capital can withstand challenges, innovate, and transition from adversity to prosperity, gradually achieving their ideals. Previous studies have also reported^(29)^ that Psychological Capital can significantly predict nurses’ Work Engagement, with more vital Psychological Capital leading to higher Work Engagement in relevant work. This study investigated 1400 emergency and critical care nurses and has a larger sample than Percunda and Putri’s study; his study just has 165 nurses.

Furthermore, the study finds that Psychological Capital positively correlates with Psychological Resilience among emergency and critical care nurses. This means that when nurses perceive higher Psychological Capital, their Psychological Resilience is more robust. Psychological Capital may enhance individuals’ sense of trust and belonging, enhancing their ability to adapt to adverse environments^(30)^.In one study^(31)^, group-based Psychological Capital intervention programs-delivered through structured training sessions and workshops focusing on self-efficacy, hope, optimism, and resilience—effectively improved ICU nurses’ Psychological Resilience, enhanced their emotional state, and reduced occupational burnout.

These results are consistent with a multi-province study, which found that nurses’ well-being influences turnover intention through the serial mediation of Psychological Capital and Work Engagement, underscoring PsyCap’s central role in shaping positive work behaviors^(32)^. Our findings further demonstrate that, in emergency and critical care settings, Psychological Resilience is a key pathway linking Psychological Capital to Work Engagement. Similarly, a recent study showed that psychological capital appreciation mediates the relationship between resilience and burnout among ICU nurses. Compared with that study, the present findings extend the evidence by showing that Psychological Resilience also acts as a mediator between Psychological Capital and Work Engagement, highlighting its dual role in reducing negative outcomes and enhancing positive work attitudes^(17)^.

### The Contribution of This Study to the Development of the Nursing Field

Emergency and critical care nurses work in demanding environments characterized by high stress, heavy workloads, and significant risks, making them particularly vulnerable to psychological fatigue, occupational burnout, and work-related stress. This study demonstrates that nurses with higher Psychological Capital exhibit stronger adaptability when confronting workplace challenges, as well as enhanced self-efficacy, hope, and optimism. Furthermore, Psychological Resilience partially mediates the relationship between Psychological Capital and Work Engagement, suggesting that fostering resilience can help nurses better manage stress and enhance engagement. Significant differences in Psychological Capital, Psychological Resilience, and Work Engagement were observed across nurses with different age groups, levels of experience, and educational backgrounds. These findings emphasize the need for tailored psychological support strategies based on individual characteristics. Higher Psychological Capital was also associated with stronger Work Engagement, indicating that career development planning, positive reinforcement strategies, and role identity education may cultivate a stronger sense of professional pride and belonging among nurses. Together, these insights provide valuable guidance for hospital administrators and nursing managers in establishing a systematic and comprehensive approach to mental health management for emergency and critical care nurses.

## LIMITATIONS AND FUTURE RESEARCH

This study has a few limitations that should be considered. First, the findings are based on a cross-sectional design, which means we cannot establish causal relationships between Psychological Capital, Work Engagement, and Psychological Resilience. Future research should consider qualitative studies, longitudinal studies, and intervention studies. Additionally, the reliance on self-reported data may introduce biases, such as social desirability bias, that could affect the accuracy of the findings. Future research should consider longitudinal designs to track changes in Psychological Capital, resilience, and Work Engagement over time.

## CONCLUSION

This study clarifies the relationships among Psychological Capital, Work Engagement, and Psychological Resilience among emergency and critical care nurses and confirms the mediating role of Psychological Resilience. The results demonstrate significant positive correlations among Psychological Capital, Work Engagement, and Psychological Resilience. Psychological Capital was found to be a significant predictor of Work Engagement, such that higher levels of Psychological Capital were associated with stronger work engagement among nurses. In addition, nurses with higher Psychological Capital were better able to cope with professional and social challenges, thereby enhancing their adaptability and Psychological Resilience. Importantly, Psychological Resilience partially mediated the relationship between Psychological Capital and Work Engagement, exerting both direct effects on Work Engagement and indirect effects through Psychological Capital.

## Data Availability

The entire dataset supporting the results of this study is available upon request to the corresponding author.
