# Supplementary material for: The impact of psychological capital on emergency and critical care nurses’ work engagement: the mediating effect of psychological resilience
Source: Rev Esc Enferm USP. 2026 Apr 3;60:e20250290. doi: 10.1590/1980-220X-REEUSP-2025-0290en (PMC13089683; doi:10.1590/1980-220X-REEUSP-2025-0290en)
Supplement: Translation

# Translation: El impacto del capital psicológico en el compromiso laboral de las enfermeras de urgencias y cuidados críticos: el efecto mediador de la resiliencia psicológica

**DOI:** 10.1590/1980-220X-REEUSP-2025-0290es

**Authors:** Yuyan Wu, Kecai Xu, Peili Xu, Yefei Wang, Chaoliang Tang

**Affiliations:** 1 University of Science and Technology of China, Division of Life Sciences and Medicine, The First Affiliated Hospital of University of Science and Technology of China, Department of Anesthesiology, Hefei, Anhui Province, China.; 2 Anhui Provincial Cancer Hospital, Department of Anesthesiology, Hefei, Anhui Province, China.; 3 Anhui Medical University, The Third Affiliated Hospital of Anhui Medical University, Department of Nursing, Hefei, Anhui Province, China.; 4 AnHui Institute of Medicine, School of Nursing, Hefei, Anhui Province, China.

**Keywords:** Compromiso Laboral, Resiliencia Psicológica, Análisis de Mediación

## Abstract

**Objetivo:**

Este estudio aborda esta brecha examinando cómo el Capital Psicológico impacta directamente en el Compromiso Laboral y cómo la Resiliencia Psicológica media esta relación entre el personal de enfermería de urgencias y cuidados críticos.

**Método:**

Este estudio empleó un diseño de encuesta transversal, utilizando un muestreo aleatorio para seleccionar a 1400 personal de enfermería de urgencias y cuidados críticos de 23 hospitales en China. Se solicitó a los participantes que completaran cuestionarios validados que medían el Capital Psicológico, la Resiliencia Psicológica y el Compromiso Laboral. Los datos se analizaron mediante la correlación de Pearson y el modelo de ecuaciones estructurales (SEM) para evaluar los efectos directos de la Resiliencia Psicológica y sus efectos mediadores en la relación entre el Capital Psicológico y el Compromiso Laboral.

**Resultados:**

Los resultados mostraron que el Capital Psicológico influye positivamente en el Compromiso Laboral entre el personal de enfermería de urgencias y cuidados críticos. Además, la Resiliencia Psicológica desempeña un papel mediador significativo en esta relación, lo que sugiere que el personal de enfermería con mayor Capital Psicológico está más comprometido con su trabajo, en parte debido a su mayor resiliencia.

**Conclusión:**

Este estudio proporciona información valiosa para mejorar la salud mental y el Compromiso Laboral del personal de enfermería de urgencias y cuidados críticos.

## INTRODUCCIÓN

Dada la naturaleza exigente de su entorno de trabajo, las enfermeras de las unidades de urgencias y cuidados intensivos (UCI) están constantemente en primera línea de la práctica médica. En comparación con sus homólogas de otros departamentos, soportan niveles sustancialmente más altos de estrés relacionado con el trabajo, lo que a menudo conduce al agotamiento y a elevadas tasas de rotación^(1)^. El capital psicológico comprende rasgos psicológicos positivos como la autoeficacia, la esperanza y el optimismo, que influyen significativamente en el comportamiento y el rendimiento de las personas^(2)^. El capital psicológico se considera una cualidad psicológica avanzada, un recurso psicológico y un estado psicológico positivo que promueve el desarrollo personal y la mejora del rendimiento. Las personas con un alto capital psicológico pueden soportar los retos, innovar y transformar la adversidad en oportunidad, logrando gradualmente sus ideales^(3)^. Los estudios han demostrado que el capital psicológico influye en el comportamiento y las actitudes de los empleados, determinando su inteligencia emocional y motivándolos a pesar de las influencias negativas^(4)^.

El compromiso laboral está influenciado por varios factores, entre los cuales el capital psicológico, un concepto psicológico emergente, ha recibido una amplia atención en los últimos años^(5)^. El compromiso laboral es un estado mental positivo y satisfactorio, tanto emocional como cognitivo, que se caracteriza por el vigor, la dedicación y la absorción^(6)^. Es un indicador clave de las actitudes y comportamientos de las enfermeras^(7)^. Las investigaciones^(8)^ indican que las enfermeras de cuidados intensivos en China muestran niveles de compromiso laboral de moderados a bajos. Este compromiso insuficiente compromete la calidad de la atención de enfermería y aumenta las intenciones de rotación, lo que supone un riesgo significativo para la retención de profesionales de enfermería cualificados^(9)^. 

La resiliencia psicológica se refiere a mantener emociones y comportamientos estables ante el estrés, los retos y la adversidad, adaptándose gradualmente, afrontando, recuperándose e incluso creciendo a partir de estas experiencias. Incluye perspectivas orientadas a los rasgos, a los resultados y a los procesos. La resiliencia orientada a los rasgos es la capacidad de una persona para manejar el estrés, los reveses y los acontecimientos adversos de la vida^(10)^. La resiliencia orientada a los resultados considera que las personas que se adaptan bien después de experiencias de alto riesgo tienen una buena resiliencia psicológica^(11)^. La resiliencia orientada a los procesos la enfatiza como un proceso dinámico que implica factores protectores y diferencias individuales a la hora de afrontar el estrés y los reveses^(12)^. Por lo tanto, la resiliencia psicológica puede ayudar a las enfermeras a mantener una mentalidad positiva y altos niveles de compromiso laboral cuando se enfrentan a retos y estrés en el trabajo^(13)^. Las investigaciones sugieren que las enfermeras con una fuerte resiliencia psicológica están mejor preparadas para manejar la adversidad y el trauma durante las emergencias de salud pública, gracias a su capacidad de automotivarse^(14)^.

El estudio reveló que la satisfacción laboral, el capital psicológico y el compromiso laboral de las enfermeras estaban significativamente interrelacionados, y que el capital psicológico mostraba una fuerte y directa correlación positiva con el compromiso laboral^(15)^. Numerosas investigaciones han demostrado que existe un fuerte vínculo entre el capital psicológico y la resiliencia psicológica. Entre las enfermeras recién contratadas, aquellas con un capital psicológico más bajo pero una mayor resiliencia psicológica tienden a mostrar una mayor capacidad de adaptación a la hora de manejar emergencias^(16)^. Además, se ha demostrado que las intervenciones dirigidas al capital psicológico mejoran significativamente la resiliencia psicológica de las enfermeras de la UCI, mejoran su bienestar emocional y alivian el agotamiento^(17)^. 

A nivel internacional, se han reportado retos similares. Las enfermeras de urgencias de Estados Unidos experimentan con frecuencia fatiga por compasión y agotamiento debido a las elevadas cargas de trabajo y las exigencias emocionales^(18)^. En Corea del Sur, los síntomas depresivos y la intención de rotación entre las enfermeras están estrechamente relacionados con una resiliencia inadecuada y la calidad de vida profesional^(19)^. En la investigación europea sobre enfermería, un mayor capital psicológico se ha asociado positivamente con un mayor compromiso laboral y un menor burnout, lo que sugiere su papel protector en la salud mental y el rendimiento de las enfermeras^(20)^. Una revisión sistemática confirmó además que la resiliencia es un recurso psicológico vital que permite a las enfermeras hacer frente al estrés y mantener su compromiso en diferentes sistemas de atención de salud^(21)^. Estos hallazgos ponen de relieve la relevancia global del capital psicológico y la resiliencia psicológica a la hora de configurar el compromiso laboral y reducir el agotamiento entre el personal de enfermería.

El estudio examinó las interconexiones entre el compromiso laboral, la resiliencia psicológica, el trabajo emocional y el burnout del personal de enfermería. Los resultados revelaron una fuerte asociación positiva entre el compromiso laboral y la resiliencia psicológica. Sobre la base de estas conclusiones, se recomendó aumentar la formación en resiliencia psicológica para fomentar un mayor compromiso laboral y reducir el agotamiento entre el personal de enfermería^(22)^.

Este estudio tiene como objetivo responder a las siguientes hipótesis de investigación: (1) El capital psicológico influye en el compromiso laboral entre el personal de enfermería de urgencias y cuidados intensivos. (2) El capital psicológico influye en la resiliencia psicológica entre el personal de enfermería de urgencias y cuidados intensivos. (3) La resiliencia psicológica influye en el compromiso laboral entre el personal de enfermería de urgencias y cuidados intensivos. (4) La resiliencia psicológica media la relación entre el capital psicológico y el compromiso laboral (Figura 1).

**Figura 1:** Modelo de investigación de hipótesis.

Si se responden estas preguntas, los resultados pueden proporcionar evidencia teórica para mejorar el compromiso laboral entre las enfermeras de urgencias y cuidados intensivos, así como orientación práctica para que los administradores hospitalarios desarrollen intervenciones adecuadas.

## MÉTODOS

### Diseño del Estudio

Este estudio adoptó un diseño de encuesta transversal, guiado por la lista de verificación Strengthening the Reporting of Observational Studies in Epidemiology (STROBE), que es adecuada para estudios transversales observacionales.

### Entorno del Estudio

El estudio se llevó a cabo entre enfermeras de urgencias y cuidados intensivos empleadas en 23 hospitales de toda China, incluyendo tanto instituciones públicas como privadas.

### Participantes y Muestreo

El tamaño mínimo requerido de la muestra se calculó utilizando la fórmula de Slovin $n=\frac{\text{N}}{{\text{1+Ne}}^{\text{2}}}$ , con un nivel de confianza del 95 % y un margen de error del 5 %^(23)^. Con una población finita de *N* = 1406 enfermeras de urgencias y cuidados intensivos en 23 hospitales, el tamaño mínimo requerido de la muestra era de $n=\frac{\text{1406}}{\text{1+1406}\times \text{0}{\text{.05}}^{\text{2}}}\approx 311$ . Para mejorar la representatividad, permitir análisis de subgrupos por región y tipo de hospital, y minimizar el sesgo de no respuesta, se adoptó una estrategia de censo casi completo. En consecuencia, se invitó a participar a todas las enfermeras elegibles de los 23 hospitales y se obtuvieron 1,400 respuestas válidas (99,6 % de la población objetivo). El muestreo aleatorio estratificado garantizó una representación proporcional por región y tipo de hospital.

Los criterios de inclusión fueron: (1) tener 18 años o más, (2) tener al menos un año de experiencia como enfermera de urgencias y cuidados intensivos, y (3) dar su consentimiento informado y participar voluntariamente en la encuesta.

Los criterios de exclusión fueron: (1) incapacidad para comprender correctamente el contenido del cuestionario y (2) falta de voluntad para participar activamente.

Este estudio fue aprobado por el Comité De Ética Del Primer Hospital Del Pueblo De Hefei, código ERC 2024-260-05.

### Procedimientos

Se seleccionó como sujetos de la encuesta a enfermeras de urgencias y cuidados intensivos en activo. La encuesta se realizó utilizando la plataforma Questionnaire Star^(24)^. Antes de la implementación de la encuesta, los investigadores recibieron una formación estandarizada para garantizar la correcta ejecución de los procedimientos de la encuesta y abordar posibles problemas, asegurando así la coherencia en la administración del cuestionario. Antes de distribuir la encuesta, se explicó claramente a las enfermeras el propósito del estudio y se obtuvo su consentimiento informado, asegurándose de que las participantes comprendieran el estudio y participaran voluntariamente. La encuesta se realizó en momentos que no coincidían con los periodos de mayor actividad de las enfermeras, lo que les permitió disponer de tiempo y energía suficientes para completar el cuestionario. Se mantuvo la paciencia y la profesionalidad durante todo el proceso de la encuesta y se respondieron todas las preguntas planteadas por las enfermeras. La encuesta se diseñó para completarse en 20 minutos.

### Medidas

#### Se Utilizaron Cuatro Instrumentos

En este estudio se utilizaron cuatro instrumentos: un cuestionario de información general, la Escala de Capital Psicológico, la Escala de Resiliencia Psicológica y la Escala de Compromiso Laboral.

### Información General

El cuestionario de información general fue diseñado por los investigadores y se utilizó para recolectar datos sobre el género, la edad, el estado civil, la formación académica, el título profesional, la institución empleadora y los puestos administrativos de las enfermeras.

### Capital Psicológico

El capital psicológico se evaluó utilizando la Escala de Capital Psicológico, que incluye cuatro dimensiones —optimismo, resiliencia, esperanza y autoeficacia— y consta de 20 ítems. La escala utiliza un formato Likert de seis puntos, en el que las puntuaciones más altas indican niveles más elevados de capital psicológico. Cada ítem se puntúa de 0 a 5, lo que corresponde a «Muy en desacuerdo», «En desacuerdo», «Ligeramente en desacuerdo», «Ligeramente de acuerdo», «De acuerdo» y «Muy de acuerdo». Los coeficientes alfa de Cronbach para la escala total y las cuatro dimensiones oscilan entre 0,718 y 0,923, y la fiabilidad test-retest para la escala total es de 0,819. La versión china de la escala tiene un coeficiente alfa de Cronbach de 0,898^(25)^. La escala demostró una buena validez de constructo (CMIN/DF = 13,86, CFI = 0,95, TLI = 0,94, RMSEA = 0,09, RMR = 0,05), con cargas factoriales para ítems individuales superiores a 0,76. Los valores medios de varianza extraída (AVE) para las cuatro dimensiones fueron 0,70, 0,85, 0,96 y 0,89.

### Resiliencia Psicológica

La resiliencia psicológica se evaluó utilizando la versión china de la Escala de Resiliencia de Connor-Davidson (CD-RISC), traducida y revisada por Yu y Zhang^(26)^.

La versión china de la CD-RISC consta de tres dimensiones— tenacidad, fuerza y optimismo—e incluye 25 ítems. Utiliza un sistema de puntuación de cinco puntos: 1 = «Nunca», 2 = «Rara vez», 3 = «A veces», 4 = «A menudo» y 5 = «Siempre», con un rango de puntuación total de 25 a 125. Las puntuaciones más altas indican una mayor resiliencia psicológica y una mayor resistencia al estrés. El coeficiente alfa de Cronbach para la escala total es de 0,91, lo que indica una buena fiabilidad y validez y respalda su idoneidad para evaluar la resiliencia psicológica de las enfermeras.

### Compromiso Laboral

El compromiso laboral se evaluó utilizando la versión china de la Escala de Compromiso Laboral de Utrecht (UWES-9)^(27)^. Esta escala se divide en tres dimensiones: vigor, dedicación y absorción, y consta de nueve ítems. Cada ítem se puntúa de 0 a 6 (0 = nunca a 6 = siempre), y las puntuaciones más altas indican niveles de compromiso más elevados. La validez del constructo de la escala se evaluó mediante un análisis factorial exploratorio y un análisis factorial confirmatorio. La UWES-9 demuestra una validez de constructo aceptable, con un modelo de tres factores que representa el 57,76 % de la varianza y estadísticas de ajuste satisfactorias. El alfa de Cronbach para la escala total es de 0,93, con valores alfa de 0,78, 0,80 y 0,81 para el vigor, la dedicación y la absorción, respectivamente, en poblaciones chinas.

### Control de Calidad

Se implementaron estrictas medidas de control de calidad a lo largo del proceso de investigación para garantizar el rigor científico y la precisión de los resultados del estudio. Durante la fase de diseño de la investigación, el equipo de investigación llevó a cabo una revisión de la literatura y consultó a expertos para obtener una comprensión inicial del compromiso laboral de las enfermeras de urgencias y cuidados intensivos y sus factores influyentes relacionados, finalizando así el diseño del estudio. Se seleccionaron los instrumentos de investigación adecuados en función de los objetivos del estudio y se desarrolló un plan de encuesta científicamente estandarizado a través de múltiples discusiones.

Durante la fase de encuesta formal, los cuestionarios se recolectaron, verificaron y organizaron inmediatamente después de que los participantes los completaran. En la fase de organización de datos, dos personas codificaron de forma independiente los cuestionarios válidos y verificaron dos veces los datos para garantizar su precisión. Tras la verificación, los datos se introdujeron en el software Epidata 3,1^(28)^. Después de la introducción de datos, los miembros del equipo realizaron comprobaciones lógicas de los valores de cada dimensión de los indicadores de evaluación para garantizar la precisión de los datos y la fiabilidad de los resultados del estudio.

### Análisis de Datos

Los análisis estadísticos descriptivos de las variables del estudio se realizaron utilizando SPSS 24,0. Las variables categóricas se expresaron como frecuencias y porcentajes (*n*, %), mientras que las variables continuas se expresaron como medias ± desviaciones estándar. Las comparaciones entre dos grupos se realizaron utilizando pruebas *t* de muestras independientes, y las comparaciones entre múltiples grupos se realizaron utilizando análisis de varianza (ANOVA). Se utilizó el coeficiente de correlación producto-momento de Pearson para examinar las relaciones entre las variables. Antes de realizar el modelo de ecuaciones estructurales (structural equation modeling - SEM), se llevaron a cabo la medida de Kaiser-Meyer-Olkin (KMO) y la prueba de esfericidad de Bartlett para evaluar la idoneidad de los datos para el análisis factorial. Se utilizó el SEM para examinar las relaciones entre el capital psicológico, el compromiso laboral y la resiliencia psicológica. Los análisis SEM se realizaron utilizando AMOS 22,0, lo que permitió probar tanto los modelos de medición como los estructurales. Para garantizar la solidez de los resultados, se empleó el bootstrapping con 5000 remuestreos para probar la significación de los efectos indirectos y las vías de mediación, confirmando así la fiabilidad y validez de los efectos de mediación observados.

### Consideraciones Éticas

Este estudio fue aprobado por el Comité de Ética del Primer Hospital Popular de Hefei (código ERC 2024-260-05). La participación fue voluntaria, se obtuvo el consentimiento informado y se mantuvo estrictamente la confidencialidad y el anonimato durante todo el estudio.

## RESULTADOS

### Características de la Muestra

Se obtuvieron un total de 1400 cuestionarios válidos (tasa de respuesta efectiva del 100 %), con 707 mujeres encuestadas (50,50 %) y 693 hombres encuestados (49,50 %).

En cuanto al tipo de institución, 486 encuestados (34,71 %) procedían de hospitales privados y 914 (65,29 %) de hospitales públicos. Los títulos profesionales de los encuestados eran los siguientes: 399 enfermeros (28,50 %), 442 enfermeros superiores (31,57 %), 543 supervisores de enfermería (38,79 %) y 16 jefes adjuntos de enfermería y superiores (1,14 %). En el sistema de enfermería chino, las enfermeras se encargan principalmente de la atención directa al paciente y de las tareas clínicas rutinarias; las enfermeras superiores asumen responsabilidades clínicas más avanzadas y orientan al personal subalterno; las supervisoras de enfermería se encargan de gestionar las salas o unidades, coordinar los equipos de enfermería y garantizar la calidad de la atención; mientras que las subdirectoras de enfermería y cargos superiores desempeñan funciones de alta dirección, supervisan la administración, la formación y la investigación en materia de enfermería y contribuyen a la elaboración de políticas. La distribución por edades fue la siguiente: 20-30 años (595 encuestados, 42,50 %), 31-40 años (443 encuestados, 31,64 %), 41-50 años (329 encuestados, 23,50 %) y más de 50 años (33 encuestados, 2,36 %).

En cuanto al estado civil, 1176 encuestados (84,00 %) estaban casados, 189 encuestados (13,50 %) eran solteros y 35 encuestados (2,50 %) estaban divorciados. Además, 450 encuestados (32,14 %) no ocupaban puestos administrativos, mientras que 950 encuestados (67,86 %) sí ocupaban puestos administrativos. En cuanto al nivel educativo más alto, 592 encuestados (42,29 %) tenían una licenciatura, 708 encuestados (50,57 %) tenían una maestría y 100 encuestados (7,14 %) tenían un doctorado. Los años de experiencia laboral se distribuyeron de la siguiente manera: 1-10 años (595 encuestados, 42,50 %), 11-20 años (443 encuestados, 31,64 %) y más de 20 años (362 encuestados, 25,86 %).

### Fiabilidad y Validez

Capital psicológico. La escala demostró una buena validez de constructo (CMIN/DF = 13,86, CFI = 0,95, TLI = 0,94, RMSEA = 0,09, RMR = 0,05), con una carga factorial de los ítems individuales superior a 0,76. Los valores AVE para las cuatro dimensiones fueron 0,70, 0,85, 0,96 y 0,89.

Resiliencia psicológica. La escala demostró una buena validez de constructo (CMIN/DF = 8,34; CFI = 0,95; TLI = 0,95; RMSEA = 0,07; RMR = 0,02), con una carga factorial de los ítems individuales superior a 0,69. Los valores AVE para las cuatro dimensiones fueron 0,74, 0,73 y 0,67.

Compromiso laboral. La escala demostró una validez de constructo aceptable (CMIN/DF = 47,26; CFI = 0,98; TLI = 0,94; RMSEA = 0,18; RMR = 0,01), con una carga factorial de los ítems individuales superior a 0,90. Los valores AVE para las cuatro dimensiones fueron 0,76, 0,94 y 0,88.

### Resultados de la Prueba de un Solo Factor de Harman

La encuesta se realizó de forma anónima e incluyó ítems con puntuación inversa para controlar el sesgo del método común. La prueba de un solo factor de Harman examinó los datos en busca de sesgos del método común. El análisis incluyó las variables principales de capital psicológico, compromiso laboral y resiliencia psicológica. Los resultados indicaron χ2/df = 13,24, CFI = 0,26, TLI = 0,34 y RMSEA = 0,27, lo que sugiere que no hay un sesgo grave del método estándar en los datos del estudio, lo que refuerza la credibilidad de los resultados del estudio. La prueba reveló que ningún factor único emergió como dominante, lo que sugiere que el sesgo del método común no fue una preocupación importante en este estudio.

### Estado Actual del Capital Psicológico, el Compromiso Laboral y la Resiliencia Psicológica de las Enfermeras de Urgencias y Cuidados Intensivos

Se realizaron análisis estadísticos descriptivos de las variables del estudio utilizando SPSS 24,0. Los datos categóricos se expresaron como frecuencia y porcentaje (*n*, %), mientras que los datos continuos se expresaron como media ± desviación estándar. Las comparaciones entre dos grupos se realizaron utilizando la prueba t para muestras independientes, mientras que las comparaciones entre múltiples grupos se realizaron utilizando ANOVA.

Se utilizó el coeficiente de correlación producto-momento de Pearson para examinar las relaciones entre las variables. Antes de realizar el SEM, se llevaron a cabo la medida de Kaiser-Meyer-Olkin (KMO) y la prueba de esfericidad de Bartlett para evaluar la adecuación de los datos para el análisis factorial. Los datos se analizaron utilizando SEM para examinar las relaciones entre el capital psicológico, el compromiso laboral y la resiliencia psicológica. El SEM se realizó utilizando AMOS 22,0, lo que permitió probar los modelos de medición y estructurales. 

### Análisis de Correlación

Como se muestra en la Tabla 1, se realizó el análisis de correlación para examinar las relaciones entre el capital psicológico, el compromiso laboral y la resiliencia psicológica, utilizando el coeficiente de correlación de Pearson para representar la fuerza de estas relaciones. El coeficiente de correlación entre el capital psicológico y el compromiso laboral mostró significación (*r* = 0,403, *p* < 0,01). El coeficiente de correlación entre el capital psicológico y la resiliencia psicológica también mostró significación (*r* = 0,540, *p* < 0,01). Del mismo modo, el coeficiente de correlación entre el compromiso laboral y la resiliencia psicológica fue significativo (*r* = 0,415, *p* < 0,01), lo que indica correlaciones positivas.

**Tabla 1:** Análisis de correlación entre el capital psicológico, el compromiso laboral y la resiliencia psicológica entre enfermeros de urgencias y cuidados intensivos — Hefei, Anhui, China, 2024.

	Capital psicológico	Compromiso laboral	Resiliencia psicológica
Capital psicológico	1		
Compromiso laboral	0,403**	1	
Resiliencia psicológica	0,540**	0,415**	1

Nota: *p < 0,05

**p < 0,01.

### Prueba del Efecto Mediador

Este estudio utilizó SEM para evaluar el efecto mediador de la resiliencia psicológica en la relación entre el capital psicológico y el compromiso laboral entre las enfermeras de urgencias y cuidados intensivos. El análisis se realizó con el software AMOS 22,0, aplicando el método Bootstrap percentil con corrección de sesgo y 5000 remuestreos para calcular el intervalo de confianza del 95 %. El análisis SEM controló datos generales como el género, la edad y el cargo.

Se tomó el compromiso laboral como variable dependiente, el capital psicológico como variable independiente y la resiliencia psicológica como variable mediadora. Se examinaron tanto los efectos directos como los indirectos. El modelo de mediación mostró un buen ajuste: CMIN/DF = 9,47, CFI = 0,90, TLI = 0,90, RMSEA = 0,08, RMR = 0,04. Los resultados estandarizados de cada ruta de regresión se presentan en la Figura 2. El capital psicológico predijo positivamente la resiliencia psicológica (β = 0,56, SE = 0,026, p < 0,001). La resiliencia psicológica predijo positivamente el compromiso laboral (β = 0,27, SE = 0,027, p < 0,001). El capital psicológico predijo positivamente la resiliencia psicológica (β = 0,27, SE = 0,027, *p* < 0,001). Los resultados indican que la resiliencia psicológica desempeña un papel mediador parcial significativo en la relación entre el capital psicológico y el compromiso laboral.

**Figura 2:** Resultados del análisis de mediación (*n* = 1400).

Los resultados del análisis del efecto de mediación (sin estandarizar) se muestran en la Tabla 2. El efecto directo del capital psicológico sobre el compromiso laboral se estima en 0,233, con un intervalo de confianza bootstrap del 95 % que oscila entre 0,180 y 0,286. El efecto indirecto a través del mediador «resiliencia psicológica» es de 0,132, con un intervalo que oscila entre 0,091 y 0,173. El impacto total que combina las vías directas e indirectas es de 0,365, con un intervalo de confianza de 0,272 a 0,459. Cabe destacar que el efecto de mediación representa aproximadamente el 36,23 % del impacto total, lo que pone de relieve el importante papel de la resiliencia psicológica en la mediación de la influencia del capital psicológico en el compromiso laboral.

**Tabla 2:** Resumen de los resultados de la prueba del efecto de mediación — Hefei, Anhui, China, 2024.

	Valor	95% NBIC	95% NAIC	Proporción
Efecto directo	0,233	0,180	0,286	63,8%
Efecto indirecto	0,132	0,091	0,173	36,2%
Efecto total	0,365	0,272	0,459	

Nota. NBIC = Nivel bajo de Intervalo de Confianza; NAIC = Nivel alto de Intervalo de Confianza

## DISCUSIÓN

### Estado Actual y Relaciones Entre el Capital Psicológico, el Compromiso Laboral y la Resiliencia Psicológica de las Enfermeras de Urgencias y Cuidados Intensivos

Este estudio explora la relación más profunda entre el capital psicológico y el compromiso laboral entre las enfermeras de urgencias y cuidados intensivos, considerando la resiliencia psicológica como variable mediadora. Los resultados muestran una correlación positiva entre el capital psicológico, el compromiso laboral y la resiliencia psicológica, lo que sugiere que las enfermeras de urgencias y cuidados intensivos con un mayor capital psicológico también pueden tener una mayor resiliencia psicológica y un mejor compromiso laboral. El capital psicológico es un recurso psicológico y un estado psicológico positivo que promueve el desarrollo personal y la mejora del rendimiento. El capital psicológico pertenece a las cualidades psicológicas avanzadas, incluyendo la resiliencia emocional y psicológica. Las personas con un capital psicológico vital pueden soportar los desafíos, innovar y pasar de la adversidad a la prosperidad, logrando gradualmente sus ideales. Estudios anteriores también han informado^(29)^ que el capital psicológico puede predecir significativamente el compromiso laboral de las enfermeras, ya que un capital psicológico más vital conduce a un mayor compromiso laboral en el trabajo relevante. Este estudio investigó a 1400 enfermeras de urgencias y cuidados intensivos y cuenta con una muestra más amplia que el estudio de Percunda y Putri, que solo incluye a 165 enfermeras.

Además, el estudio concluye que el capital psicológico se correlaciona positivamente con la resiliencia psicológica entre las enfermeras de urgencias y cuidados intensivos. Esto significa que cuando las enfermeras perciben un mayor capital psicológico, su resiliencia psicológica es más sólida. El capital psicológico puede mejorar el sentido de confianza y pertenencia de las personas, lo que aumenta su capacidad para adaptarse a entornos adversos^(30)^. En un estudio^(31)^, los programas de intervención de capital psicológico basados en grupos, impartidos a través de sesiones de formación estructuradas y talleres centrados en la autoeficacia, la esperanza, el optimismo y la resiliencia, mejoraron eficazmente la resiliencia psicológica de las enfermeras de la UCI, mejoraron su estado emocional y redujeron el agotamiento profesional.

Estos resultados concuerdan con un estudio realizado en varias provincias, que descubrió que el bienestar de las enfermeras influye en la intención de rotación a través de la mediación en serie del capital psicológico y el compromiso laboral, lo que subraya el papel central del capital psicológico en la configuración de comportamientos laborales positivos^(32)^. Nuestros hallazgos demuestran además que, en entornos de urgencias y cuidados intensivos, la resiliencia psicológica es una vía clave que vincula el capital psicológico con el compromiso laboral. Del mismo modo, un estudio reciente mostró que la apreciación del capital psicológico media la relación entre la resiliencia y el agotamiento entre las enfermeras de la UCI. En comparación con ese estudio, los presentes hallazgos amplían la evidencia al mostrar que la resiliencia psicológica también actúa como mediadora entre el capital psicológico y el compromiso laboral, lo que destaca su doble función en la reducción de los resultados negativos y la mejora de las actitudes laborales positivas^(17)^.

### Contribución de Este Estudio al Desarrollo del Campo de la Enfermería

Las enfermeras de urgencias y cuidados intensivos trabajan en entornos exigentes que se caracterizan por un alto nivel de estrés, una gran carga de trabajo y riesgos significativos, lo que las hace especialmente vulnerables a la fatiga psicológica, el agotamiento profesional y el estrés relacionado con el trabajo. Este estudio demuestra que las enfermeras con un mayor capital psicológico muestran una mayor capacidad de adaptación a los retos del lugar de trabajo, así como una mayor autoeficacia, esperanza y optimismo. Además, la resiliencia psicológica media parcialmente la relación entre el capital psicológico y el compromiso laboral, lo que sugiere que fomentar la resiliencia puede ayudar a las enfermeras a gestionar mejor el estrés y mejorar su compromiso. Se observaron diferencias significativas en el capital psicológico, la resiliencia psicológica y el compromiso laboral entre enfermeras de diferentes grupos de edad, niveles de experiencia y formación académica. Estos hallazgos enfatizan la necesidad de estrategias de apoyo psicológico personalizadas basadas en las características individuales. Un mayor capital psicológico también se asoció con un mayor compromiso laboral, lo que indica que la planificación del desarrollo profesional, las estrategias de refuerzo positivo y la educación sobre la identidad profesional pueden cultivar un mayor sentido de orgullo profesional y pertenencia entre las enfermeras. En conjunto, estas ideas proporcionan una valiosa orientación a los administradores de hospitales y a los directores de enfermería para establecer un enfoque sistemático y completo de la gestión de la salud mental de las enfermeras de urgencias y cuidados intensivos.

## LIMITACIONES E INVESTIGACIONES FUTURAS

Este estudio tiene algunas limitaciones que deben tenerse en cuenta. En primer lugar, los resultados se basan en un diseño transversal, lo que significa que no podemos establecer relaciones causales entre el capital psicológico, el compromiso laboral y la resiliencia psicológica. Las investigaciones futuras deberían considerar estudios cualitativos, estudios longitudinales y estudios de intervención. Además, la dependencia de los datos autoinformados puede introducir sesgos, como el sesgo de deseabilidad social, que podrían afectar a la precisión de los resultados. Las investigaciones futuras deberían considerar diseños longitudinales para seguir los cambios en el capital psicológico, la resiliencia y el compromiso laboral a lo largo del tiempo.

## CONCLUSIÓN

Este estudio aclara las relaciones entre el capital psicológico, el compromiso laboral y la resiliencia psicológica entre las enfermeras de urgencias y cuidados intensivos y confirma el papel mediador de la resiliencia psicológica. Los resultados demuestran correlaciones positivas significativas entre el capital psicológico, el compromiso laboral y la resiliencia psicológica. Se descubrió que el capital psicológico era un predictor significativo del compromiso laboral, de tal manera que los niveles más altos de capital psicológico se asociaban con un mayor compromiso laboral entre las enfermeras. Además, las enfermeras con un mayor capital psicológico eran más capaces de hacer frente a los retos profesionales y sociales, lo que mejoraba su adaptabilidad y su resiliencia psicológica. Es importante destacar que la resiliencia psicológica mediaba parcialmente la relación entre el capital psicológico y el compromiso laboral, ejerciendo tanto efectos directos sobre el compromiso laboral como efectos indirectos a través del capital psicológico.

## Data Availability

El conjunto de datos que respaldan las conclusiones de este estudio está disponible previa solicitud al autor correspondiente.
